# Early identification of mild cognitive impairment: an innovative model using ocular biomarkers

**DOI:** 10.3389/fnagi.2025.1492804

**Published:** 2025-04-22

**Authors:** Lingjing Zhang, Yanwei Wang, Yuming Liu, Zi Ye, Zhaohui Li

**Affiliations:** ^1^School of Medicine, Nankai University, Tianjin, China; ^2^Department of Ophthalmology, The General Hospital of the People’s Liberation Army, Beijing, China; ^3^Department of General Surgery, No. 926 Hospital, Joint Logistics Support Force of PLA, Kaiyuan, China; ^4^Medical School of Chinese PLA, Beijing, China

**Keywords:** mild cognitive impairment, ocular biomarkers, Alzheimer’s disease, prediction model, web-based calculator

## Abstract

**Background:**

Alzheimer’s disease (AD) is a neurodegenerative disorder characterized by progressive, irreversible brain damage. Current diagnostic procedures for AD are both costly and highly invasive for patients. Age-related cataract (ARC), a common ocular condition in elderly populations, correlates with a 1.43-fold increased risk of developing AD. This study sought to establish a novel model for early detection of mild cognitive impairment (MCI) in patients with ARC.

**Methods:**

The study prospectively collected 170 monocular data as training dataset and 65 monocular data from another independent medical center as test dataset. Demographic data and comprehensive ophthalmic examination results were collected. The least absolute shrinkage and selection operator (LASSO) method and multivariate logistic regression analysis were performed using R software for dimensionality reduction and variable selection. A nomogram was constructed, and its discriminative ability was evaluated using receiver operating characteristic (ROC) curve, area under the ROC curve (AUC) with 95% confidence interval (CI), as well as sensitivity and specificity. Internal validation was performed using 1,000-resample bootstrap analysis, while model calibration was assessed through calibration curves and Brier scores. Decision curve analysis (DCA) was performed to evaluate clinical utility. A baseline model incorporating demographic variables was developed for comparison with the nomogram. Additionally, an external dataset from an independent medical center was employed as a test set to further validate the nomogram’s predictive performance. An online calculator was created using the “DynNom” and “rsconnect” functions.

**Results:**

Through LASSO regression and multivariate logistic regression analyses, six variables were identified and incorporated into the nomogram: age (OR: 1.097; 95%CI: 1.041–1.161; *p* < 0.001), years of education (OR: 0.333; 95%CI: 0.140–0.749; *p* = 0.010), diastolic blood pressure (OR: 0.949; 95%CI: 0.907–0.990; *p* = 0.019), short posterior ciliary artery flow rate (OR: 1.063; 95%CI: 1.008–1.132; *p* = 0.038), vertical cup-to-disc ratio (OR: 11.927; 95%CI: 1.059–155.308; *p* = 0.049), and peripapillary retinal nerve fiber layer thickness (inferior; OR: 0.979; 95%CI: 0.964–0.993; *p* = 0.005). The nomogram demonstrated strong discriminatory power for the diagnosis of MCI, with the area under the ROC curve reaching 0.791 (95%CI: 0.722–0.864) in the training dataset and 0.750 (95%CI: 0.627–0.858) in the external dataset. Calibration curve validation showed good agreement between predicted and ideal probabilities (*p* > 0.05, Brier score = 0.171). DCA indicated substantial net benefit across most threshold probabilities in both training and test datasets, supporting the nomogram’s clinical utility.

**Conclusion:**

Through systematic analysis of clinical data, this study established and validated a novel online calculator for identifying early cognitive impairment in patients with ARC, using demographic and ocular biomarkers, thereby providing a visual representation of the prediction model.

## 1 Introduction

Alzheimer’s disease (AD) is the predominant cause of dementia, accounting for approximately 60-80% of cases. Its primary pathological hallmarks are β-amyloid (Aβ) plaques and neurofibrillary tangles composed of hyperphosphorylated tau, which can impair synaptic plasticity and lead to neuronal death (Begcevic et al., 2018). Clinical AD is preceded by a prolonged asymptomatic phase, termed preclinical AD, characterized by the accumulation of brain pathology, which may begin 10-20 years before cognitive symptoms emerge (Bateman et al., 2012; Ameri et al., 2020). The progression of AD includes mild cognitive impairment (MCI), characterized by cognitive decline exceeding age-appropriate and education-appropriate norms but not yet significantly affecting daily activities (Albert et al., 2011). Considering the irreversible nature of AD, early detection of MCI is crucial. Current diagnostic methods for probable AD primarily rely on neurocognitive tests, brain imaging, and cerebrospinal fluid (CSF) analysis (Veitch et al., 2022). However, these procedures are often expensive and invasive for patients. Furthermore, despite these diagnostic tools, AD diagnoses remain inaccurate in 10-15% of cases owing to limitations in sensitivity and specificity (Weller and Budson, 2018). The development of cost-effective, non-invasive biomarkers in alternative body fluids or tissues for the detection of early cognitive dysfunction remains an active research focus. Recent studies have identified blood-based biomarkers, including tau protein, neurofilament light chain (NFL), and Aβ, as potential indicators of cognitive decline in AD, although research regarding their effectiveness remains preliminary, warranting further investigation (Baiardi et al., 2022).

A comprehensive retrospective cohort study from the Taiwan National Health Insurance Program established that elderly individuals with cataracts exhibit a 1.43-fold increased risk of developing AD (Lai et al., 2014). Light deficiency caused by cataracts may influence this process by disrupting the suprachiasmatic nucleus (SCN) regulation of circadian rhythms, subsequently exacerbating age-related conditions such as depression, insomnia, and cognitive impairment (Moncaster et al., 2022). This suggests that age-related cataract (ARC), being an age-associated condition, may predispose individuals to AD. Recent research has increasingly focused on the eye, specifically the retina, as an accessible window into brain function. Anatomically and developmentally, the retina originates from pluripotent ectodermal cells of the developing diencephalon neuroectoderm and exhibits numerous structural and functional similarities with brain tissue (Lamb et al., 2007). Initial observations of ocular symptoms in patients with AD were reported by Schlotterer et al. in 1984 (Schlotterer et al., 1984). Subsequently, in 1986, Hinton et al. first documented histological evidence of retinal abnormalities in AD, including substantial loss of retinal ganglion cell neurons, decreased thickness of the retinal nerve fiber layer (RNFL), and optic nerve degeneration (Hinton et al., 1986). Given these findings, ocular biomarkers present promising opportunities for early identification of AD through non-invasive and multi-modal approaches. Therefore, this study aimed to develop and validate a prediction model to facilitate early identification of MCI in patients with ARC who demonstrate risk factors for the progression of AD.

## 2 Materials and methods

### 2.1 Patient selection

This prospective study enrolled 170 patients (60 male, 110 female; mean age 71 years) with ARC awaiting cataract surgery from the Ophthalmology Unit of the First Medical Center of Chinese PLA General Hospital between November 1, 2023, and June 30, 2024, forming the training dataset. Additionally, 65 individuals meeting the same criteria were enrolled from the Ophthalmology Unit of the Third Medical Center of Chinese PLA General Hospital between October 1, 2024, and January 20, 2025, constituting the test dataset. These medical centers operate independently in terms of patient care. One eye from each participant was randomly selected for the study.

The inclusion criteria encompassed: (1) cataract diagnosis confirmed via slit-lamp biomicroscopic lens examination; (2) complete clinical data availability; and (3) normal basic functional independence. The exclusion criteria comprised: (1) severe cataracts or uncooperative status affecting examination quality; (2) concurrent fundus pathologies, including glaucoma, age-related macular degeneration, diabetic retinopathy, optic neuropathy, high myopia, and demyelinating disease; (3) conditions potentially causing cognitive impairment apart from AD, such as Parkinson’s disease, multiple sclerosis, anxiety disorders, depressive disorders, hypothyroidism, and vitamin deficiency; and (4) personal or three-generation familial psychiatric history.

The Medical Ethics Committee of the Chinese PLA General Hospital approved the study protocol (S2024-160-01). Written informed consent was obtained from all participants prior to study enrollment.

### 2.2 Neuropsychological assessments

**FIGURE 1:**
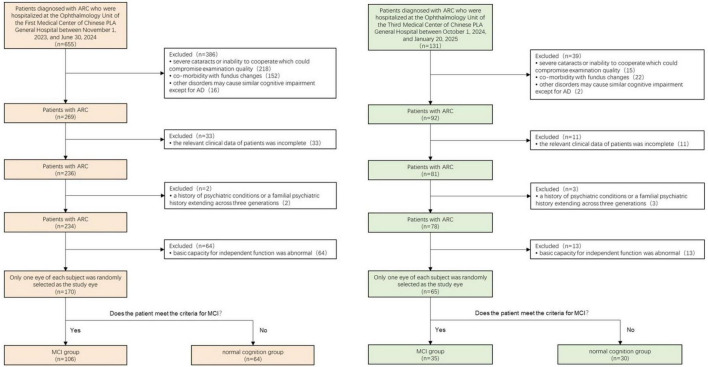
Schematic representation of the selection process of the training and test datasets.

All participants underwent comprehensive neuropsychological assessments across multiple domains: (1) The Montreal Cognitive Assessment (MoCA) scale evaluated cognitive function, with an additional point allocated to participants with 12 years or less of education (MoCAadj); (2) Basic Activities of Daily Living (BADL) scale and Instrumental Activities of Daily Living (IADL) scale assessed daily activity capabilities; (3) Global Deterioration Scale (GDS) evaluated cognitive impairment symptoms and stages; and (4) Hamilton Anxiety Scale/Hamilton Depression Scale and Neuropsychiatric Inventory (NPI) assessed neuropsychiatric symptoms. MCI diagnoses adhered to Petersen’s diagnostic criteria (Petersen, 2004). The inclusion criteria comprised: (1) self-reported memory loss persisting for 3 months or longer; (2) MoCAadj score < 26; (2) intact BADL and normal or minimally impaired IADL; and (3) absence of dementia, defined as a Global Deterioration Scale score of 2 or 3. Based on these criteria, participants were classified into the MCI group and the normal cognition group. The screening procedure is illustrated in Figure 1.

### 2.3 Data collection

All baseline characteristics and medical histories were obtained from the hospital’s electronic medical records. Each patient underwent a comprehensive ophthalmic evaluation comprising slit-lamp examination, refraction assessment, visual acuity testing with best-corrected visual acuity measurement (using a standardized Snellen eye chart), intraocular pressure measurement (via non-contact tonometry), dilated fundus examination (using binocular indirect ophthalmoscopy), spectral domain-optical coherence tomography (SD-OCT), optical coherence tomography angiography (OCTA), and retrobulbar blood flow examination.

#### 2.3.1 OCT and OCTA examinations

OCT and OCTA examinations were conducted using an SD-OCT system and the AngioVue system on the Optovue RTVue XR Avanti (Optovue-100, Fremont, CA, United States). The scanning parameters included a speed of 26,000 scans per second, optical axial resolution of 5 μm, and 6 mm × 6 mm scanning patterns. During the examination, participants were positioned at an appropriate distance from the instrument and instructed to maintain focus on a central fixation point throughout the procedure. Mydriasis was not required. All examinations were performed by an experienced physician.

**FIGURE 2:**
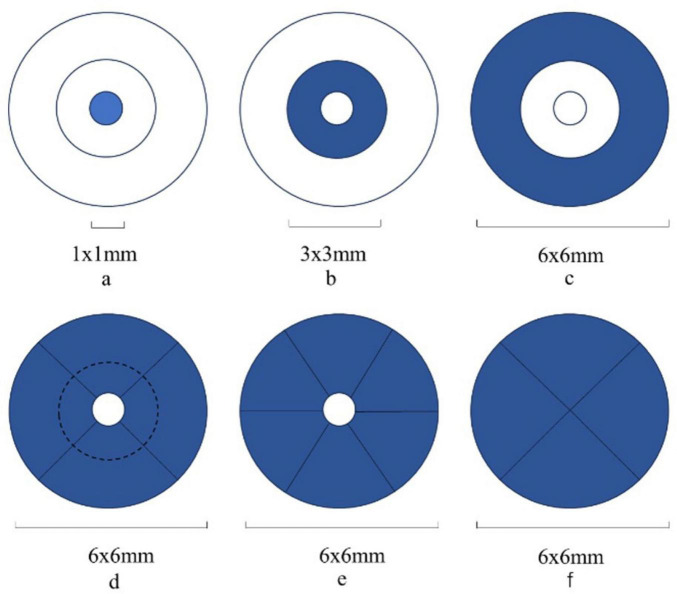
**(a–f)** Schematic diagrams of regional division of OCT and OCTA examination.

**FIGURE 3:**
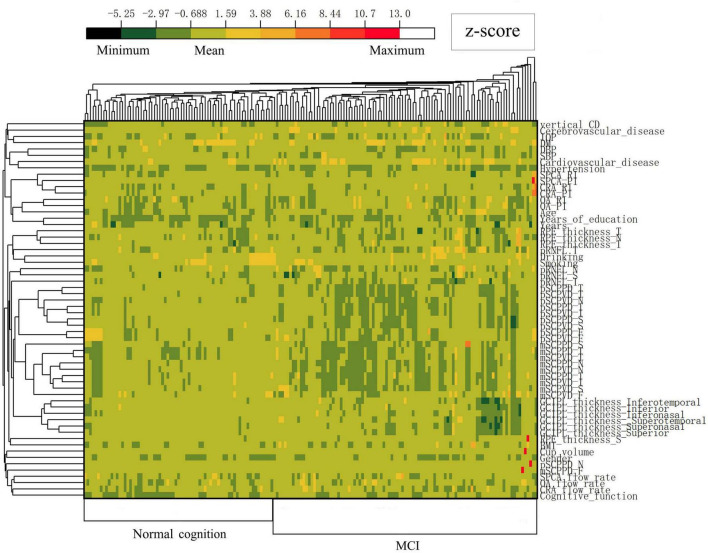
Heatmap illustrating standardized variables (z-scores by row) in the normal cognition group and MCI group of ARC patients. Colors closer to red and dark green represent higher and lower z-scores, respectively.

**FIGURE 4:**
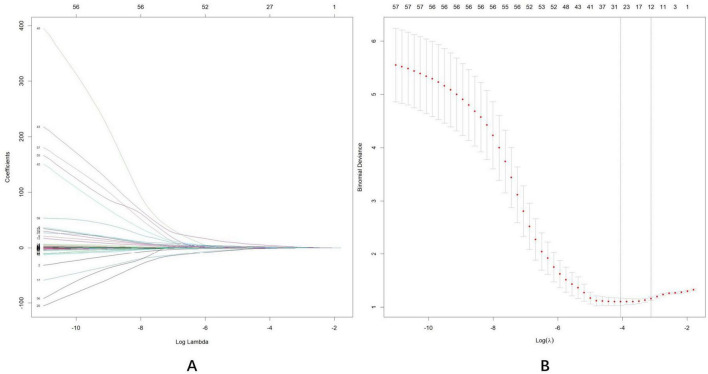
Least absolute shrinkage and selection operator (LASSO) regression analysis was used for data dimensionality reduction and variable selection. **(A)** LASSO coefficient profiles of the58 candidate predictors. **(B)** Five-fold cross validation with a minimum error criterion was performed to determine the optimal penalization estimate of λ in LASSO regression.

The OCT acquisition protocol consisted of several measurements: (1) retinal thickness (measured from the inner limiting membrane [ILM] to the retinal pigment epithelium [RPE], ILM-RPE thickness) included macular central subfield thickness in the central circular area of 1 mm diameter (Figure 2a) and four quadrants (superior [S], inferior [I], temporal [T], and nasal [N]) in the circular area of 6 mm diameter, excluding the macular central subfield (Figure 2d); (2) macular ganglion cell-inner plexiform layer thickness (mGC-IPL thickness) divided the 6 mm diameter circular area, excluding the macular central subfield, into six quadrants (superior [S], inferior [I], superonasal [SN], inferonasal [IN], superotemporal [ST], and inferotemporal [IT]) (Figure 2e); (3) peripapillary retinal nerve fiber layer thickness (pRNFL thickness) divided the 6 mm diameter circular area into four quadrants:(superior [S], inferior [I], temporal [T], and nasal [N]) (Figure 2f); (4) optic nerve parameters included the disc area, rim area, cup volume, and cup-to-disc ratio (C/D) in both vertical and horizontal dimensions.

OCTA scans were utilized to analyze vessel density (VD) and perfusion density (PD) of the superficial capillary plexus (SCP). The analysis incorporated the following distinct areas: (1) inner ring: a central circular region with 1 mm diameter (Figure 2a); (2) middle ring: a circular region of 3 mm diameter, excluding the inner ring (Figure 2b); (3) outer ring: a circular region of 6 mm diameter, excluding both the inner and middle rings (Figure 2c); (4) quadrant division: the circular region of 6 mm diameter, excluding the inner ring, divided into four quadrants (superior [S], inferior [I], temporal [T], and nasal [N]) (Figure 2d).

#### 2.3.2 Retrobulbar blood flow examination

Retrobulbar blood flow was assessed using the Acuson Sequoia 512 diagnostic ultrasound system (SIEMENS, Germany) by a qualified ultrasound physician with ≥ 3 years of experience in retrobulbar blood flow sonography. Participants were examined in a supine position with closed eyes. The arterial flow velocity, pulsatility index, and resistance index (RI) were measured in retrobulbar vessels, including the ophthalmic artery, central retinal artery (CRA), and short posterior ciliary artery (SPCA).

### 2.4 Statistical analysis

The pattern of missing data was evaluated using the md.pattern() function from the “mice” package in R software to determine the missing data mechanism. Multiple imputation was implemented to address missing data, with 10 imputations performed following standard recommendations under the Missing At Random (MAR) mechanism. The Multiple Imputation by Chained Equations approach was applied using the “mice” package in R software. Clustering heat maps were generated using Origin 2021 software to standardize the dataset by rows through z-score normalization. This process involves calculating the difference between each data point and the mean of the respective row, which is then divided by the adjusted standard deviation of that row. Expressed as: *z* = (x-μ)/σ.

Data normality was assessed using the Shapiro–Wilk test in SPSS version 27.0. Normally distributed data were presented as mean ± standard deviation and analyzed using Student’s *t*-test. Non-normally distributed data were expressed as median (interquartile range; IQR) and compared using the Mann–Whitney *U* test. Categorical variables were presented as percentages, with between-group differences evaluated using the chi-squared test or Fischer’s exact test.

Least absolute shrinkage and selection operator (LASSO) regression analysis was employed for data dimensionality reduction and variable selection by using the “glmnet” and “MASS” packages in R software. In this process, we implemented the glm (family = binomial) function to build a binary classification model. Before applying LASSO, we standardized the continuous predictive factors (Mean = 0, SD = 1) to prevent the influence of scale differences of variables. Non-significant variables were eliminated by increasing the penalty coefficient λ, which was determined through 5-fold cross-validation based on cv.glmnet function. Subsequently, multivariable logistic regression analysis was performed to further refine variable selection based on LASSO regression results. A nomogram was constructed and visualized using the “rms” package, incorporating the identified independent risk factors.

A baseline model incorporating demographic variables was established and compared with the complete nomogram model to assess improvements in predictive capability following the inclusion of ocular indicators. The evaluation utilized three distinct metrics: receiver operating characteristic (ROC) curve, Net Reclassification Index (NRI), and Integrated Discrimination Improvement (IDI), implemented through the “rms,” “nricens,” and “PredictABEL” packages in R software. Model discrimination was assessed using ROC curves with 95% confidence intervals, calculated via the “fbroc” package. The model’s sensitivity, specificity, positive predictive value (PPV), negative predictive value (NPV), positive likelihood ratio (PLR), and negative likelihood ratio (NLR) were computed using the “reportROC” package. The optimal diagnostic threshold was determined by maximizing Youden’s index, calculated as Youden’s index = sensitivity + specificity – 1.

Model validation included internal verification through 1,000-resample bootstrap analysis, while calibration curves and Brier scores assessed the goodness of fit in the training dataset. Clinical utility was evaluated using decision curve analysis (DCA). Additionally, an external validation dataset from an independent medical center served as a test set to further evaluate the nomogram’s predictive performance.

The online calculator was developed using the nomogram formula through the “DynNom” and “rsconnect” functions and hosted at https://www.shinyapps.io/. All statistical analyses and online calculator construction were performed using R software (version 4.4.0),^1^ SPSS version 27.0 (IBM Corp., Armonk, NY, United States), and Origin 2021. A two-sided *P* < 0.05 was considered statistically significant.

## 3 Results

### 3.1 Population characteristics

The training dataset included 170 monocular data points that met the exclusion and inclusion criteria. Based on MCI diagnostic criteria, participants were categorized into 64 (37.6%) cases of normal cognition and 106 (62.4%) cases of MCI. Table 1 presents part of the general characteristics of the training dataset. The test dataset comprised 65 monocular data points, consisting of 30 (46.2%) cases of normal cognition and 35 (53.8%) cases of MCI. (Complete data for both training and test datasets are available in Supplementary Material 1.) A clustering heat map was generated to evaluate the potential of these variables as diagnostic markers for MCI risk, illustrating the expression characteristics of variables in both the normal cognition and MCI groups of patients with ARC (Figure 3). The horizontal axis of the heatmap represents individual patient samples, with the left side corresponding to the normal group and the right side to the MCI group. The vertical axis denotes the study indicators. As illustrated in the figure, the MCI group displays a greater enrichment of darker colors, suggesting that certain indicators are more pronounced in the affected group and may serve as potential predictors of the disease.

**TABLE 1:** Summary of variables in the training dataset.

Predictive variables	MCI (*n* = 106)	Normal cognition (*n* = 64)	*P*
**Demographic data**			
Age, years	73.19 ± 7.64	68.52 ± 7.42	<0.001
**Gender, n (%)**			0.846
Male	38 (35.8)	22 (34.4)	
Female	68 (64.2)	42 (65.6)	
SBP, mmHg	140 (134.00,148.25)	140 (132.25, 149.25)	0.261
DBP, mmHg	79 (71.75, 83.00)	84.5 (75.75, 89.00)	0.001
**Years of education, n (%)**			0.186
≤ 12 years	64 (60.4)	32 (50.0)	
>12 years	42 (39.6)	32 (50.0)	
Smoking, n (%)	21 (19.8)	8 (12.5)	0.220
Drinking, n (%)	16 (15.1)	8 (12.5)	0.638
IOP, mmHg	13.95 (12.00, 16.00)	14.00 (12.23, 16.00)	0.700
**Comorbidities**			
Hypertension, n (%)	52 (49.1)	30 (46.9)	0.783
Diabetes, n (%)	24 (22.6)	20 (31.3)	0.214
Cardiovascular disease, n (%)	22 (20.8)	10 (15.6)	0.407
Cerebrovascular disease, n (%)	12 (11.3)	4 (6.3)	0.417
Anxiety, n (%)	5 (4.7)	2 (3.1)	0.712
Depression, n (%)	4 (3.8)	1 (1.6)	0.651
**Retrobulbar blood flow**			
SPCA flow rate, cm/s	20.40 (16.98, 26.70)	19.30 (14.03, 23.30)	0.036
SPCA PI	1.77 (1.49, 2.00)	1.66 (0.96, 1.66)	0.133
SPCA RI	0.81 (0.74, 0.86)	0.79 (0.61, 0.79)	0.162
**OCT of macula**			
ILM-RPE thickness (T), μm	286.00 (272.38, 292.38)	280.50 (267.50, 292.88)	0.306
mGCIPL thickness (S), μm	81.00 (71.75, 85.00)	78.00 (72.00, 84.00)	0.186
**OCTA of macula**			
mSCPVD(T), mm^–1^	9.63 (4.71, 15.23)	9.35 (3.41, 14.51)	0.226
mSCPPD(T), mm^–1^	0.23 (4.71, 15.23)	0.21 (0.08, 0.34)	0.141
**OCT of optic disc**			
vertical C/D	0.51 (00.3.44, 0.63)	0.49 (0.38, 0.57)	0.039
Cup volume, mm^3^	0.12 (0.04, 0.25)	0.09 (0.05, 0.30)	0.988
pRNFL(I), μm	105.00 (89.75, 127.00)	119.00 (105.25, 133.00)	0.001
pRNFL(T), μm	70.00 (60.75, 77.25)	69.00 (58.75, 74.00)	0.311
**OCTA of optic disc**			
pSCPVD(T), mm^–1^	14.15 (8.49, 16.73)	15.05 (10.09, 16.89)	0.744
pSCPPD(T), mm^–1^	0.33 (0.18, 0.41)	0.35 (0.21, 0.40)	0.804

SBP, systolic blood pressure; DBP, diastolic blood pressure; IOP, intraocular pressure; SPCA, short posterior ciliary artery; PI, pulsatility index; RI, resistance index; ILM -RPE (T), inner limiting membrane to the retinal pigment epithelium (temporal); mGCIPL (S), macular ganglion cell-inner plexiform layer thicknes (superior); mSCPVD (T), the vessel density of macular superficial capillary plexus (temporal); mSCPPD (T), the perfusion density of macular superficial capillary plexus (temporal); C/D, cup-to-disc ratio; pRNFL(I/T), peripapillary retinal nerve fiber layer (inferior/temporal); pSCPVD (T), the vessel density of peripapillary superficial capillary plexus (temporal); pSCPPD (T), the vessel density of peripapillary superficial capillary plexus (temporal). OCT, optical coherence tomography; OCTA, optical coherence tomography angiography.

### 3.2 LASSO regression analysis

Initially, 58 associated variables were entered into the LASSO regression algorithm using 5-fold cross-validation. Through the incremental adjustment of the penalty coefficient (λ), non-significant variables were systematically eliminated (Figures 4A,B). When λmin (λ = 0.014) was selected, the number of variables was up to 31. Therefore, we further increased the λ to λ_1se (λ = 0.045), where the number of variables was 12. It can not only ensure the performance of the model, but also prevent the model from becoming too cumbersome due to the inclusion of too many variables. The list of λ values considered during cross-validation is available in appendix. The analysis identified 12 potential variables with non-zero coefficients: age (β, 0.188; *p* < 0.001), years of education (β, −1.454; *p* = 0.007), diastolic blood pressure (DBP) (β, −0.082; *p* = 0.002), CRA flow rate (β, 0.151; *p* = 0.024), CRA RI (β, −1.656; *p* = 0.384), SPCA flow rate (β, 0.071; *p* = 0.036), ILM-RPE thickness (T) (β, 0.038; *p* = 0.002), mSCPPD (T) (β, 5.856; *p* = 0.002), vertical C/D (β, 3.566; *p* = 0.030), cup volume (β, −0.096; *p* = 0.723), pRNFL (I) (β, −0.036; *p* = 0.001), pRNFL (T) (β, 0.048; *p* = 0.012). Subsequently, two variable with *p* > 0.05 was excluded: CRA RI (*p* = 0.384), cup volume (*p* = 0.723). While previous studies indicated a negative correlation between ILM-RPE thickness (T), mSCPPD (T), and pRNFL (T) biomarkers and cognitive impairment (Ge et al., 2021), our analysis revealed positive coefficients for these variables, contradicting previous findings. Additionally, population characteristics analysis demonstrated no statistically significant differences among these three variables (*p* > 0.05). Considering that nomogram models operate optimally with 4–7 variables, these three variables were excluded from the final model.

### 3.3 Multivariate logistic regression analysis

The initially screened variables were incorporated into a multivariate regression analysis to identify independent factors predictive of MCI. Table 2 presents the β coefficients, standard error (SE), odds ratios (OR), 95% confidence intervals (95% CI), and *P-*values of the seven variables. Subsequently, one variable with p > 0.05 was eliminated: CRA flow rate (*p* = 0.122). The final model identified six independent predictors of MCI: age (OR: 1.097; 95%CI: 1.041–1.161; *p* < 0.001), years of education (OR: 0.333; 95%CI: 0.140–0.749; *p* = 0.010), DBP (OR: 0.949; 95%CI: 0.907–0.990; *p* = 0.019), SPCA flow rate (OR: 1.063; 95%CI: 1.008–1.132; *p* = 0.038), pRNFL (I) (OR: 0.979; 95%CI: 0.964–0.993; *p* = 0.005), vertical C/D (OR: 11.927; 95%CI: 1.059–155.308; *p* = 0.049).

**TABLE 2:** Multivariate logistic regression analysis.

Intercept and variables	β	SE	OR (95% CI)	*P*
Age	0.093	0.028	1.097 (1.041–1.161)	<0.001*
Years of education	−1.101	0.425	0.333 (0.140–0.749)	0.010*
DBP	−0.052	0.022	0.949 (0.907–0.990)	0.019*
CRA flow rate	0.088	0.057	1.092 (0.980–1.226)	0.122
SPCA flow rate	0.061	0.030	1.063 (1.008–1.132)	0.038*
pRNFL(I)	−0.021	0.008	0.979 (0.964–0.993)	0.005*
Vertical C/D	2.479	1.262	11.927 (1.059–155.308)	0.049*

DBP, diastolic blood pressure; CRA, central retinal artery; SPCA, short posterior ciliary artery; pRNFL(I), peripapillary retinal nerve fiber layer (inferior); C/D, cup-to-disc ratio;

*P < 0.05.

### 3.4 Construction of nomogram and online calculator

**FIGURE 5:**
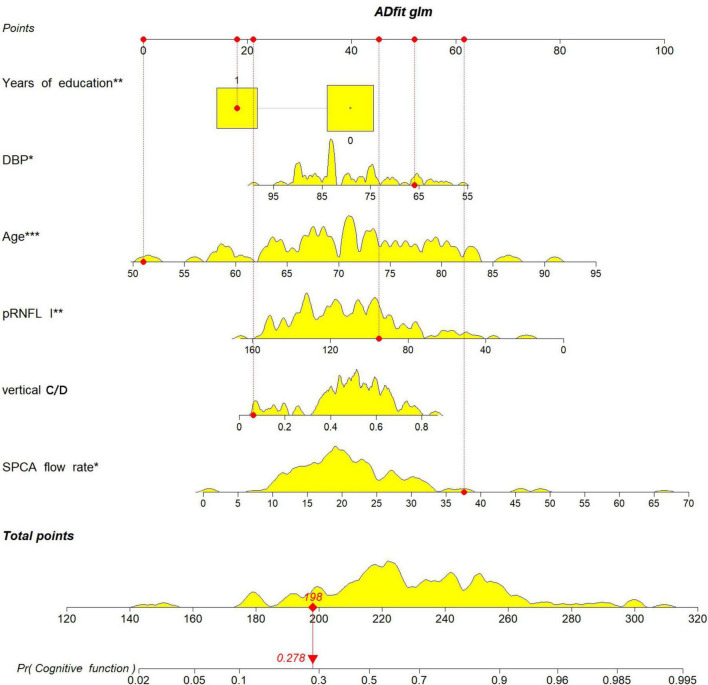
The nomogram was constructed to predict the probability of an ARC patient developing MCI by incorporating the 5 independent predictors: age, years of education, DBP, SPCA flow rate, pRNFL (I), vertical C/D.

**FIGURE 6:**
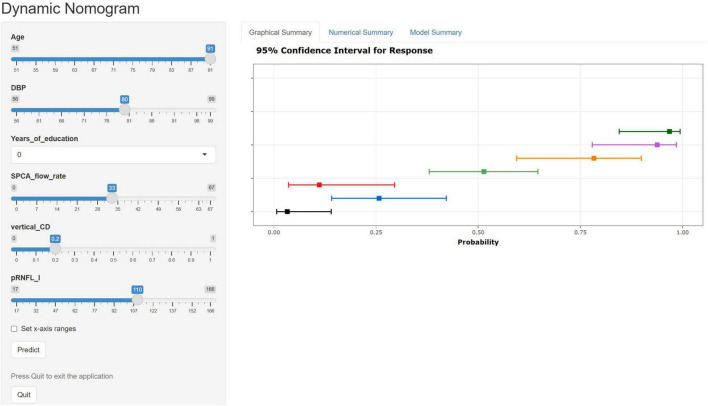
Web-based calculator web page using dynamic nomogram. We entered 7 samples into the web calculator as an example. The error bars represent the 95% confidence interval for the predicted probabilities.

A nomogram was constructed using the “rms” package to predict the probability of MCI development in patients with ARC by incorporating six independent predictors: age, years of education, DBP, SPCA flow rate, pRNFL (I), and vertical C/D, (Figure 5). The model’s methodology involved calculating the total score (bottom ruler) by summing the individual prediction index scores (top ruler), with the corresponding probability indicating the risk of MCI diagnosis. Higher total scores correlated with an increased risk of MCI. To enhance the clinical application, a web-based calculator was developed based on the dynamic nomogram using the “DynNom” and “rsconnect” functions at https://www.shinyapps.io/. This prediction model calculator is freely accessible at https://phccalculate.shinyapps.io/dynnomapp/. The user interface of the web-based calculator is shown in Figure 6. We have included 7 cases as examples for display in the calculator. The specific data, along with the actual predictor values and prediction results, is presented in Supplementary Material 1.

### 3.5 Evaluation and validation of the nomogram

**FIGURE 7:**
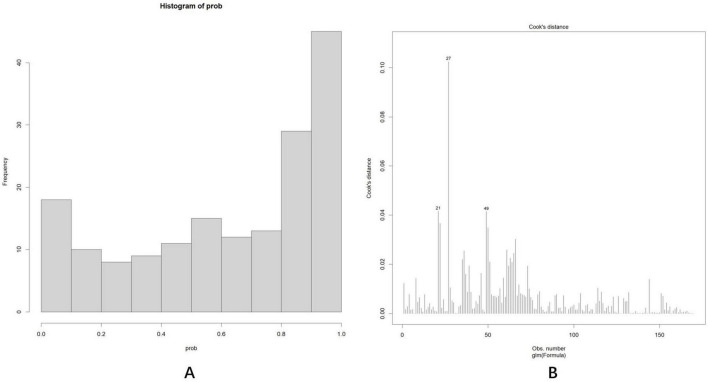
**(A)** The predicted probability of all cases was between 0 and 1, with a bipolar distribution. **(B)** Cook’s distance (Dennis Cook et al., 1977) of each sample fell within the normal range (Di < 0.5).

As shown in Figure 7A, the predicted probability of all cases ranged between 0 and 1, demonstrating appropriate differentiation. Subsequently, Cook’s distance (Dennis Cook et al., 1977) was calculated for each sample (Figure 7B), and all values remained within the normal range (Di < 0.5), indicating that no outliers significantly influenced the overall effect sizes or significance levels. Variance inflation factors (vifs) were calculated for each predictor to detect multiple collinearities, with all values approximating 1: age (vif, 1.14), years of education (vif, 1.28), DBP (vif, 1.09), SPCA flow rate (vif, 1.10), pRNFL(I) (vif, 1.08), vertical C/D (vif, 1.07).

**FIGURE 8:**
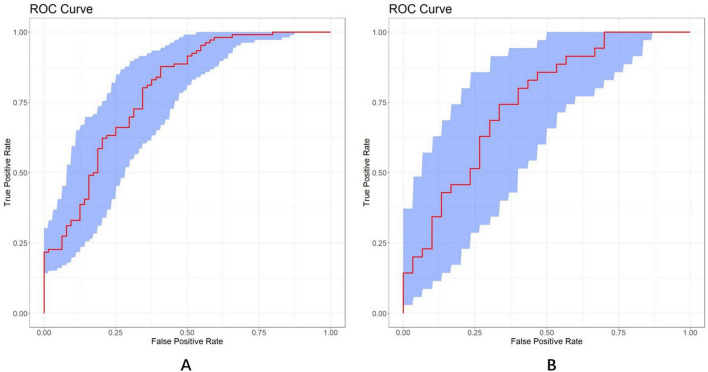
ROC curves of the LASSO model in the training **(A)** and test **(B)** dataset, respectively (AUC = 0.791 vs. 0.750). A total of 1,000 bootstrap resamples used to calculate a relatively corrected AUC and 95% CI. The blue area represents the 95% CIs. ROC, receiver operator characteristics; LASSO, least absolute shrinkage and selection operator; AUC, area under the curve.

**FIGURE 9:**
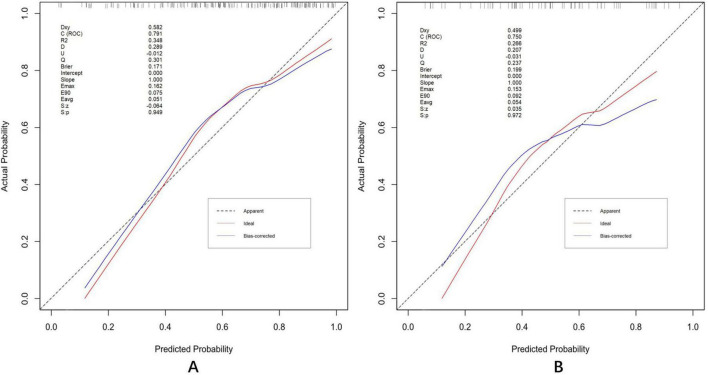
Calibration plots in the training **(A)** and test **(B)** datasets. Validation of the calibration curves exhibited good concordance between the predicted probability and ideal probability in the training dataset (*P* = 0.949) and test dataset (*P* = 0.972). *P* > 0.05 indicates a good calibration with no difference between the ideal probability and predicted probabilities.

The discrimination and calibration of the nomogram in the training and test datasets are illustrated in Figures 8A,B, respectively. Through bootstrapping validation, the area under the ROC curve values for the model were determined to be 0.791 (95%CI: 0.722–0.864) in the training dataset and 0.750 (95%CI: 0.627–0.858) in the test dataset. The 95% CIs of the calibration belt in both groups did not cross the diagonal bisector line, indicating acceptable concordance performance of the prediction model. The analysis yielded the following metrics using the “reportROC” package: sensitivity (0.877, 95%CI: 0.815–0.940), specificity (0.594, 95%CI: 0.473–0.714), PPV (0.782, 95%CI: 0.707–0.856), NPV (0.745, 95%CI: 0.625–0.865), PLR (2.16, 95%CI: 1.593–2.929), and NLR (0.207, 95%CI: 0.119–0.357). The optimal diagnostic threshold was determined using the maximum Youden’s Index, calculated as sensitivity + specificity − 1. The best cutoff value of 0.504 indicated optimal predictive performance at this decision threshold. Following 1,000 bootstrap self-sampling internal validation, the calibration curve demonstrated strong concordance between predicted and ideal probabilities in both the training dataset (P = 0.949) and test dataset (*P* = 0.972; Figures 9A,B). The Brier scores measuring prediction accuracy were 0.171 and 0.199, respectively, confirming the model’s strong probabilistic predictions.

### 3.6 Value of ocular indicators to nomogram

**FIGURE 10:**
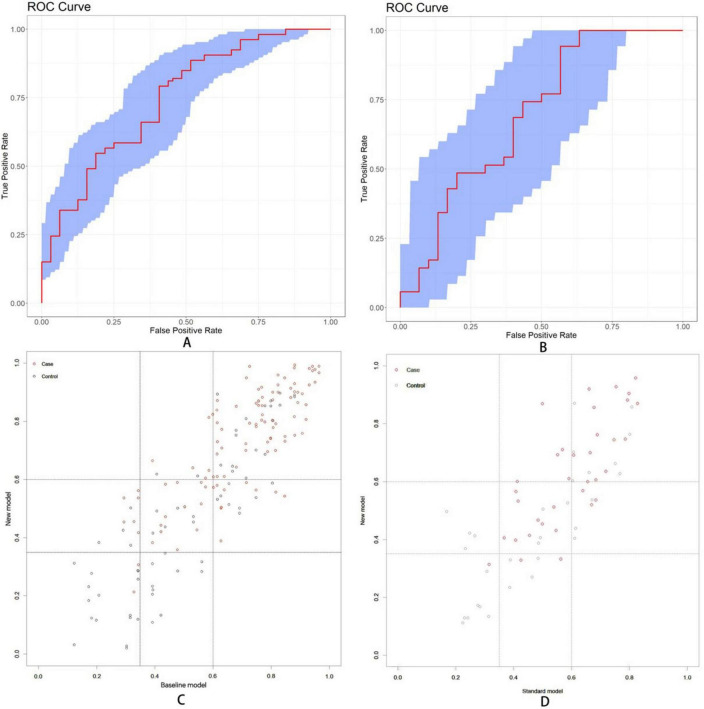
**(A)** The area under the ROC curve values for baseline model were found to be 0.746 (95% CI: 0.664-0.821) in training dataset. **(B)** The area under the ROC curve was 0.694 (95%CI: 0.554-0.828) in test dataset. **(C)** Scatter diagram in training dataset. **(D)** Scatter diagram in test dataset. Setting the lower threshold at 0.35 and the upper threshold at 0.6 effectively differentiates between the normal group and the MCI group.

For baseline analysis, a model incorporating three demographic variables (years of education, DBP, and age) was constructed in training and test datasets. This model was then compared with the complete nomogram model to assess improvements in predictive capability following the inclusion of ocular indicators. Three evaluation metrics were used, implemented through the “rms,” “nricens,” and “PredictABEL” packages in R software. In training dataset, the area under the ROC curve (Figure 10A) for the baseline model was 0.746 (95%CI: 0.664–0.821), while the complete prediction model achieved an AUC of 0.791 (95%CI: 0.722–0.864), representing a statistically significant difference (*z* = 2.107, *p* = 0.035). Subsequently, threshold values of 0.35 and 0.6 were applied as the lower and upper bounds for the prediction model using the “nricens” package, as illustrated in Figures 10C,D. NRI and IDI calculations, performed using the PredictABEL package, are presented in Table 3. Both categorical and continuous NRI, along with IDI, showed statistically significant improvements (*p* < 0.05), indicating that the complete model demonstrated enhanced predictive capacity across risk categories, continuous risks, and overall risks compared to the baseline model. In test dataset, AUC of the baseline model (Figure 10B) was 0.694 (95% CI: 0.554–0.828), while the AUC of the complete prediction model was 0.750 (95% CI: 0.627–0.858), with no statistically significant difference between the two (*p* > 0.05). NRI and IDI were also calculated, and the results showed that the IDI had a statistically significant difference (*p* < 0.05), while the NRI did not exhibit a significant difference (*p* > 0.05) (Figure 10).

**TABLE 3:** Complete model compared to baseline model.

Items	Improvement training test		95% CI training test		*P* training test	
NRI (categorical)	0.197	0.071	0.029–0.365	−0.212–0.354	0.022	0.621
NRI (continuous)	0.433	0.429	0.130–0.736	−0.038–0.895	0.005	0.072
IDI	0.076	0.071	0.036–0.115	0.007–0.136	<0.001	0.029

NRI, Net Reclassification Index, IDI, Integrated Discrimination Improvement.

### 3.7 Clinical usefulness assessment

**FIGURE 11:**
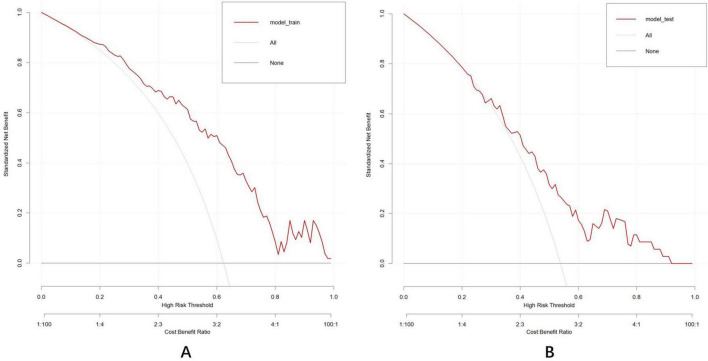
Decision curve analysis established by the training dataset **(A)** and test dataset **(B)**. The DCA curve indicated that the models contributed high net benefit in almost all threshold probabilities in both datasets. “All201D curve (gray dashed line): Assume that all individuals are at high risk, which means adopting the most aggressive intervention strategy. “None” curve (black solid line): assume that all individuals are at low risk, meaning no intervention measures are taken.

DCA was conducted to evaluate the clinical utility of the nomogram (Figures 11A,B). In the DCA curves, the ordinate (Y-axis) depicts the net benefit while the abscissa (X-axis) represents the threshold probability. The analysis revealed that the model yielded substantial net benefit across nearly all threshold probabilities in both the training and test datasets, indicating that the nomogram demonstrated considerable clinical value.

## 4 Discussion

With demographic shifts, an increasing number of individuals with low vision or blindness are experiencing cataracts (Flaxman et al., 2017). Currently, cataract surgery remains the only effective treatment, characterized by brief operative time and rapid postoperative recovery, enabling the development of ambulatory day surgery (Lawrence et al., 2015). Consequently, cataract surgery is increasingly performed in outpatient clinics rather than inpatient departments, owing to its efficacy, safety, and cost-effectiveness. However, patients with ARC are typically older and face a higher risk of concomitant cognitive dysfunction. The limited time available for communication during day surgery requires enhanced coordination. Effective management of these patients, particularly through early identification of cognitive impairment, is crucial to predict their coordination levels during the operation.

This study introduces a novel visual and user-friendly nomogram prediction model utilizing non-invasive and readily obtainable ocular biomarkers designed for the early identification of cognitive decline risk among patients with ARC. The nomogram incorporates six independent prognostic factors: age, years of education, DBP, SPCA flow rate, pRNFL (I), and vertical C/D, demonstrating significant discriminative ability in the training dataset (AUC: 0.791, 95%CI: 0.722–0.864) and the test dataset (AUC: 0.750, 95%CI: 0.627–0.85) to differentiate between individuals with MCI and those with normal cognitive function among patients with ARC, as assessed using the bootstrap method. Further validation through DCA confirmed the nomogram’s strong consistency and clinical utility.

In the training dataset, the inclusion of ocular predictors enhanced overall prediction accuracy, proving more effective than the baseline model. In the test dataset, only the IDI demonstrated a statistically significant difference, while the area under the ROC curve (AUC) and NRI showed no significant differences compared to baseline model. On the one hand, this may be related to the insufficient sample size of the test set. On the other hand, this discrepancy may be attributed to the distinct aspects emphasized by different metrics. IDI can sensitively capture continuous improvements in individual risk prediction without relying on classification thresholds, whereas the NRI measures the reclassification ability of a model under predefined risk thresholds and depends on the setting of these thresholds. Additionally, if the number of correctly upgraded cases in the event group and incorrectly upgraded cases in the non-event group offset each other, the difference in NRI might also fail to reach significance. AUC reflects overall discriminative ability but may be insensitive to localized improvements in predicted probabilities (e.g., within intermediate risk ranges). The statistical significance of IDI indicates that the new model achieved substantive improvements in the absolute accuracy of predicted probabilities or risk gradients. However, these improvements were not translated into enhanced classification capability (as reflected by NRI) or overall discriminative performance (as measured by AUC). In clinical practice, the model incorporating ocular indicators may still hold practical value for applications such as personalized risk assessment. We have reviewed pertinent literature and found that there is currently no existing model utilizing ophthalmology-related indicators to predict the risk of MCI, which highlights the innovative nature of our work. Similar type of work includes a cross-sectional study based on machine learning algorithms to predict MCI in older adults dominated by Song et al. (2025). In their study, 6,434 older adults were enrolled based on the data of the China Health and Elderly Care Longitudinal Survey (CHARLS) in 2020. Six machine learning (ML) algorithms were employed in this study: logistic regression, K-nearest neighbors (KNN), support vector machine (SVM), decision tree (DT), LightGBM, and random forest (RF). These algorithms identified five key characteristics for predicting MCI: educational level, social engagement, gender, relationship with children, and age. Some of these findings align with those of our own study. Ultimately, the area under the ROC curve for each model ranged from 0.71 to 0.77. In a study conducted by Yuan et al. focused on the early identification and support of individuals at risk of developing cognitive impairment following traumatic brain injury, several significant independent predictive factors were identified, including age, years of education, pulmonary infection status, epilepsy status, cerebrospinal fluid leakage status, and the Helsinki score (Yuan et al., 2025). Additionally, a nomogram was developed and translated into an online risk calculator, akin to the approach taken in our study.

Aging is a complex and progressive process characterized by systematic changes occurring over decades (Yin et al., 2015). Research has established that aging leads to metabolic dysregulation, insomnia, depression, and cognitive decline (Rettberg et al., 2016). Age serves as a fundamental catalyst in the development of AD (National Institute on Aging, 2015). This condition is linked to glucose hypometabolism, disrupted cholesterol homeostasis, mitochondrial dysfunction, altered immune and inflammatory responses, Aβ processing, white matter deterioration, and reduced regenerative capacity (Cai and Jeong, 2020; Klosinski et al., 2015; Masters et al., 2015; Shaw et al., 2013). This study revealed a significant positive correlation between advanced age and increased cognitive impairment risk (OR: 1.097; 95%CI: 1.041–1.161; *p* < 0.001), which aligned with previous research findings. Whitley et al. (2016) examined five cognitive measures in a large, representative UK population sample of over 40,000 individuals aged 16-100 years. The results demonstrated that all measured cognitive functions showed an earlier decline beginning around age 60.

Research indicates that educational attainment may serve as a protective factor against cognitive decline, commonly termed cognitive reserve. Education can increase regional cortical thickness in healthy individuals, contributing to increased brain reserve, while also enabling patients with AD to better manage brain atrophy effects through enhanced cognitive reserve (Liu et al., 2012). However, the cognitive benefits of education may differ across demographic characteristics such as gender and ethnicity (Sortsø et al., 2017). Additionally, education’s influence on cognitive function is closely linked to vascular pathology and appears most significant during early disease phases, highlighting the intricate relationship between education, brain health, and cognitive outcomes (Zieren et al., 2013). This study demonstrated the protective role of higher education in cognitive function among patients with ARC (OR: 0.333; 95%CI: 0.140–0.749; *p* = 0.010).

The risk of MCI showed a negative correlation with DBP (OR: 0.949; 95%CI: 0.907–0.990; *p* = 0.019). Longitudinal studies have supported these findings, suggesting that a mild to moderate increase in DBP could reduce the risk of developing AD (Verghese et al., 2003; Yang et al., 2011). A potential explanation for this relationship is that lower DBP may be insufficient for maintaining adequate cerebral perfusion, potentially contributing to cerebral Aβ accumulation (Li et al., 2022). During later life stages, increased arterial stiffness manifests through decreased DBP and elevated SBP. Prolonged exposure to elevated pulse pressure may lead to cerebral white matter damage, brain atrophy, and deterioration of cortical connections, subsequently affecting cognitive function (Del Pinto et al., 2021). Alternatively, this phenomenon might suggest that dementia onset could influence the central regulation of blood pressure, resulting in lower DBP (Masoli and Delgado, 2021). Further research has emphasized the connection between DBP and hippocampal volume, indicating that elevated DBP significantly correlates with increased hippocampal volume, potentially influencing cognitive health (Ngwa et al., 2018). Additionally, blood pressure variability, including DBP fluctuations, has been linked to cognitive outcomes. Studies on visit-to-visit blood pressure variability have shown that increased DBP variability correlates with higher risks of MCI and probable dementia, emphasizing the importance of BPV monitoring in clinical practice (Guo et al., 2023).

Since the initial documentation of AD pathology in the retina of patients with AD in the 1980s (Hinton et al., 1986), substantial evidence has emerged clarifying the relationship between retinal changes and AD. Advancements in ophthalmic technologies, particularly SD-OCT, have significantly improved resolution compared to time domain OCT, enabling detailed examination of all retinal layers in patients with AD. Research has identified a gradient of retinal thickness reduction, with more pronounced thinning in the inner retinal layers compared to the outer layers (Asanad et al., 2019). Studies across diverse populations have indicated a potential correlation between inner retinal thickness and cognitive function. MRI studies in non-demented individuals have revealed a possible connection between GC-IPL thickness and temporal and occipital lobe atrophy (Ong et al., 2015). Additionally, RNFL thinning has been associated with brain alterations in visual and limbic networks (Ong et al., 2015; Méndez-Gómez et al., 2018). While most studies indicate that retinal layers change progressively with AD progression, some research reports show no statistically significant differences (Asanad et al., 2019; Chan et al., 2019; Kesler et al., 2011; Knoll et al., 2016; Tao et al., 2019). These discrepancies may result from variations in exclusion criteria, cognitive assessment methodologies, and handling of confounding factors. In our univariate analysis, neither ILM-RPE thickness nor GC-IPL thickness showed significant differences between the MCI and normal groups (*p* > 0.05). However, pRNFL(I) thinning emerged as a risk factor for MCI (OR: 0.979; 95%CI: 0.964–0.993; *p* = 0.005). The findings suggest that pRNFL thinning precedes changes in other retinal layers, potentially indicating neurodegeneration of the central nervous system. Postmortem analyses of AD retinas have demonstrated cellular shrinkage, swelling, and vacuolization (Cheung et al., 2017; Dehghani et al., 2018). Optic disk pallor was observed even in early AD stages, attributed to axonal loss and perfusion alterations (Bambo et al., 2015). A meta-analysis indicated that a higher cup-to-disc ratio, lower height variation contour, lower rim area, and lower rim volume measured by scanning laser ophthalmoscopy may facilitate the diagnosis of AD (Ge et al., 2021). Our OCT study findings confirmed an enlarged cup-to-disc ratio as a significant risk factor for MCI (OR: 11.927; 95%CI: 1.059–155.308; *p* = 0.049).

The retinal vasculature shares structural and functional similarities with the cerebral vasculature. Research indicates that alterations in blood flow parameters precede neuronal loss (Javaid et al., 2016). This finding suggests that changes in retinal blood vessels could reflect underlying cerebrovascular pathology. The ophthalmic artery, originating from the internal carotid artery, supplies blood to the ocular region and gives rise to the central retinal artery and posterior ciliary arteries. The short posterior ciliary artery branches within the choroid, forming a choroidal vascular network that supplies the choroid, macula, and the outer retinal layers, while the central retinal artery delivers blood to the inner retina (Baldoncini et al., 2019). In this study, patients with ARC demonstrated an increased SPCA flow rate, which correlated with a higher likelihood of developing cognitive impairment (OR: 1.063; 95%CI: 1.008–1.132; *p* = 0.038). Furthermore, OCTA analysis of the superficial capillary plexus revealed no significant differences in SCP-VP and SCP-DP of the macular and optic disc regions between the MCI group and the normal group (*p* > 0.05). A study by Yoon et al. comparing patients with MCI to those with normal cognitive function found no significant differences in VD, PD, vein diameters, and the area of the foveal avascular zone (FAZ) (Yoon et al., 2019), aligning with our findings. Diverse perspectives exist on this matter (Chua et al., 2020). Based on our findings, we hypothesize that during the progression from cognitive normality to early cognitive impairments, changes in blood flow parameters of ocular supply vessels precede alterations in the retinal capillary network and retinal structure. These modifications in ocular blood flow initially arise from feedback mechanisms, such as increased arterial flow velocity. Current research exploring the relationship between ocular arteries and cognitive function remains limited. However, as components of the systemic vasculature, ocular arteries have been implicated in optic nerve damage in glaucoma, associated with changes in ocular artery blood flow and hypertension (Waliszek-Iwanicka et al., 2010). This suggests that alterations in ocular blood flow may impair visual function through effects on the optic nerve (e.g., enlarged cup-to-disc ratio), potentially contributing to cognitive-related issues.

Although recent research strongly suggests a correlation between ocular biomarkers and cognitive impairment, no definitive diagnostic model has been established. This study introduces a new nomogram prediction model based on six indicators obtained through non-invasive, accessible, safe, and cost-effective methods. The nomogram demonstrates robust performance in internal and external verification, making it applicable to diverse patient populations. Additionally, our study provides a visual representation beneficial for clinicians, particularly in basic hospitals with limited resources. This study has several limitations that warrant consideration. First, owing to the heterogeneity of OCT/OCTA devices in clinical practice, variations in image analysis software may affect readouts. Hence, this calculator applies only in settings using the OptoVue device. Second, certain potential confounding factors (such as ApoE genotype) were not fully incorporated, potentially affecting the final results. Third, the limited sample size may affect the generalizability of the results. Furthermore, the cross-sectional study design precludes causal inferences. Future large-scale cohort studies are necessary to validate these findings and conclusions.

## 5 Conclusion

This research culminates in the development of an online calculator that enables the identification of early cognitive dysfunction in patients with ARC through ocular biomarkers. The validation results confirm the model’s robust discrimination ability. The identification of novel ocular biomarkers represents a significant advancement in the assessment and management of cognitive impairment for both patients and healthcare providers.

## Data Availability

The raw data supporting the conclusions of this article will be made available by the authors, without undue reservation.

## Footnotes

1
https://www.R-project.org
