# Supplementary material for: Early identification of mild cognitive impairment: an innovative model using ocular biomarkers
Source: Front Aging Neurosci. 2025 Apr 22;17:1492804. doi: 10.3389/fnagi.2025.1492804 (PMC12052757; doi:10.3389/fnagi.2025.1492804)
Supplement: Supplementary file 1 [file Data_Sheet_1.docx]

**Supplementary Table 1:** Summary of predictive variables in the training dataset.

| Predictive variables | MCI  （*n*=106） | normal cognition  （*n*=64） | *P* |
| --- | --- | --- | --- |
| **Demographic data**  Age, years | 73.19±7.64 | 68.52±7.42 | ＜0.001 |
| Gender, n (%)  Male  Female  BMI, kg/m^2^  SBP,mmHg  DBP,mmHg  Years of education，n (%)  ≤12 years  ＞12 years  Smoking,n(%)  Drinking,n(%)  IOP,mmHg  **Comorbidities**  Hypertension,n(%)  Diabetes,n(%)  Cardiovascular disease,n(%)  Cerebrovascular disease,n(%)  Anxiety,n(%)  Depression,n(%)  **Retrobulbar blood flow**  OA flow rate,cm/s  OA PI  OA RI  CRA flow rate,cm/s  CRA PI  CRA RI  SPCA flow rate,cm/s  SPCA PI  SPCA RI  **OCT of macula**  ILM-RPE thickness(S),μm  ILM-RPE thickness(I),μm  ILM-RPE thickness(N),μm  ILM-RPE thickness(T),μm  mGCIPL thickness(S),μm  mGCIPL thickness(SN),μm  mGCIPL thickness(IN),μm  mGCIPL thickness(I),μm  mGCIPL thickness(IT),μm  mGCIPL thickness(ST),μm  **OCTA of macula**  mSCPVD(inner ring)，mm^-1^  mSCPVD(S)，mm^-1^  mSCPVD(I)，mm^-1^  mSCPVD(N)，mm^-1^  mSCPVD(T)，mm^-1^  mSCPPD(inner ring)，mm^-1^  mSCPPD(S)，mm  mSCPPD(I)，mm^-1^  mSCPPD(N)，mm^-1^  mSCPPD(T)，mm^-1^  **OCT of optic disc**  vertical C/D  Cup volume, mm^3^  pRNFL(S),μm  pRNFL(I),μm  pRNFL(N),μm  pRNFL(T),μm  **OCTA of optic disc**  pSCPVD(inner ring)，mm^-1^  pSCPVD(S)，mm^-1^  pSCPVD(I)，mm^-1^  pSCPVD(N)，mm^-1^  pSCPVD(T)，mm^-1^  pSCPPD(inner ring)，mm^-1^  pSCPPD(S)，mm^-1^  pSCPPD(I)，mm^-1^  pSCPPD(N)，mm^-1^ | 38（35.8）  68（64.2）  24.20（22.08，26.73）  140（134.00,148.25）  79（71.75，83.00）  64（60.4）  42（39.6）  21（19.8）  16（15.1）  13.95（12.00，16.00）  52（49.1）  24（22.6）  22（20.8）  12（11.3）  5(4.7)  4(3.8)  30.20（24.95，39.38）  2.03（1.66，2.63）  0.84（0.76，0.89）  11.55（9.60，14.60）  2.21（1.80，2.60）  0.86（0.77，0.97）  20.40（16.98，26.70）  1.77（1.49，2.00）  0.81（0.74，0.86）  298.50（284.38，306.50）  286.50（276.50，297.63）  206.00（291.00，316.00）  286.00（272.38，292.38）  81.00（71.75，85.00）  81.50（71.75，87.00）  79.00（69.75，83.00）  75.00（65.00，80.00）  78.50（70.00，83.00）  78.00（71.00，84.25）  0.50（0.10，2.80）  10.50（5.34，14.85）  10.40（6.04，15.85）  10.53（5.90，16.26）  9.63（4.71，15.23）  0.01（0，0.06）  0.25（0.12，0.37）  0.25（0.14，0.37）  0.25（0.10，0.36）  0.23（4.71，15.23）  0.51（00.3.44，0.63）  0.12（0.04，0.25）  107.50（88.00，127.00）  105.00（89.75，127.00）  67.00（55.00，72.00）  70.00（60.75，77.25）  2.95（1.80，4.40）  17,38（13.35，18.70）  16.13（12.25，18.20）  15.20（10.16，17.70）  14.15（8.49，16.73）  0.07（0.04，0.11）  0.43（0.32，0.48）  0.41（0.31，0.46）  0.38（0.23，0.45） | 22（34.4）  42（65.6）  24.05（21.85，26.23）  140（132.25,149.25）  84.5（75.75，89.00）  32（50.0）  32（50.0）  8（12.5）  8（12.5）  14.00（12.23，16.00）  30（46.9）  20（31.3）  10（15.6）  4（6.3）  2(3.1)  1(1.6)  26.60（22.63，33.73）  2.11（1.93，2.26）  0.84（0.79，0.88）  10.30（9.20，12.20）  2.10（1.83，2.46）  0.85（0.79，0.98）  19.30（14.03，23.30）  1.66（0.96，1.66）  0.79（0.61，0.79）  292.25（283.00，304.00）  287.25（279.00，296.88）  305.00（288.25，313.88）  280.50（267.50，292.88）  78.00（72.00，84.00）  78.00（70.00，85.75）  77.00（69.50，81.00）  74.00（68.25，79.00）  78.00（73.00，83.75）  79.00（75.00，84.75）  0.75（0.20，2.98）  10.67（7.14，14.69）  10.48（6.93，14.71）  12.55（9.40，16.19）  9.35（3.41，14.51）  0.01（0，0.04）  0.24（0.13，0.35）  0.23（0.15，0.37）  0.27（0.19，0.37）  0.21（0.08，0.34）  0.49（0.38，0.57）  0.09（0.05，0.30）  112.00（102.25，119.75）  119.00（105.25，133.00）  66.00（59.00，74.00）  69.00（58.75，74.00）  2.90（1.65，6.28）  17.70（14.11，18.99）  17.10（15.21，18.45）  15.70（10.70，17.79）  15.05（10.09，16.89）  0.07（0.04，0.17）  0.45（0.35，0.48）  0.43（0.37，0.47）  0.38（0.25，0.46） | 0.846  0.695  0.261  0.001  0.186  0.220  0.638  0.700  0.783  0.214  0.407  0.417  0.712  0.651  0.062  0.740  0.926  0.011  0.504  0.632  0.036  0.133  0.162  0.319  0.624  0.640  0.306  0.186  0.076  0.550  0.748  0.667  0.651  0.801  0.600  0.427  0.200  0.226  0.832  0.793  0.656  0.719  0.141  0.039  0.988  0.126  0.001  0.361  0.311  0.384  0.833  0.268  0.894  0.744  0.392  0.633  0.247  0.834 |
| pSCPPD(T)，mm^-1^ | 0.33（0.18，0.41） | 0.35（0.21，0.40） | 0.804 |

BMI = body mass index; SBP = systolic blood pressure; DBP = diastolic blood pressure; IOP = intraocular pressure; OA = ophthalmic artery; CRA = central retinal artery; SPCA =short posterior ciliary artery; PI = pulsatility index; RI = resistance index; OCT= optical coherence tomography; OCTA= optical coherence tomography angiography;

ILM -RPE (S/I/N/T) = inner limiting membrane to the retinal pigment epithelium (superior/inferior/ nasal/temporal); mGCIPL (S/SN/IN/I/IT/ST) = macular ganglion cell-inner plexiform layer thicknes (superior/superonasal/inferonasal/inferior/inferotemporal/superotemporal); mSCPVD (S/I/N/T) = the vessel density of macular superficial capillary plexus (superior/inferior/ nasal/temporal); mSCPPD (S/I/N/T) = the perfusion density of macular superficial capillary plexus (superior/inferior/ nasal/temporal); C/D= cup-to-disc ratio; pRNFL(S/I/N/T) = peripapillary retinal nerve fiber layer (superior/inferior/ nasal/temporal); pSCPVD (S/I/N/T) = the vessel density of peripapillary superficial capillary plexus (superior/inferior/ nasal/temporal); pSCPPD (S/I/N/T) = the vessel density of peripapillary superficial capillary plexus (superior/inferior/ nasal/temporal).

**Supplementary Table 2:** Summary of predictive variables in the test dataset:

| Predictive variables | MCI  （*n*=35） | normal cognition  （*n*=30） | *P* |
| --- | --- | --- | --- |
| **Demographic data**  Age, years | 69.67 (6.28) | 72.94 (7.21) | 0.055 |
| Gender, n (%)  Male  Female  BMI, kg/m^2^  SBP,mmHg  DBP,mmHg  Years of education，n (%)  ≤12 years  ＞12 years  Smoking,n(%)  Drinking,n(%)  IOP,mmHg  **Comorbidities**  Hypertension,n(%)  Diabetes,n(%)  Cardiovascular disease,n(%)  Cerebrovascular disease,n(%)  Anxiety,n(%)  Depression,n(%)  **Retrobulbar blood flow**  OA flow rate,cm/s  OA PI  OA RI  CRA flow rate,cm/s  CRA PI  CRA RI  SPCA flow rate,cm/s  SPCA PI  SPCA RI  **OCT of macula**  ILM-RPE thickness(S),μm  ILM-RPE thickness(I),μm  ILM-RPE thickness(N),μm  ILM-RPE thickness(T),μm  mGCIPL thickness(S),μm  mGCIPL thickness(SN),μm  mGCIPL thickness(IN),μm  mGCIPL thickness(I),μm  mGCIPL thickness(IT),μm  mGCIPL thickness(ST),μm  **OCTA of macula**  mSCPVD(inner ring)，mm^-1^  mSCPVD(S)，mm^-1^  mSCPVD(I)，mm^-1^  mSCPVD(N)，mm^-1^  mSCPVD(T)，mm^-1^  mSCPPD(inner ring)，mm^-1^  mSCPPD(S)，mm  mSCPPD(I)，mm^-1^  mSCPPD(N)，mm^-1^  mSCPPD(T)，mm^-1^  **OCT of optic disc**  vertical C/D  Cup volume, mm^3^  pRNFL(S),μm  pRNFL(I),μm  pRNFL(N),μm  pRNFL(T),μm  **OCTA of optic disc**  pSCPVD(inner ring)，mm^-1^  pSCPVD(S)，mm^-1^  pSCPVD(I)，mm^-1^  pSCPVD(N)，mm^-1^  pSCPVD(T)，mm^-1^  pSCPPD(inner ring)，mm^-1^  pSCPPD(S)，mm^-1^  pSCPPD(I)，mm^-1^  pSCPPD(N)，mm^-1^ | 15 (50.00)  15 (50.00)  140.00 [130.75, 145.00]  140（134.00,148.25）  83.10 (8.02)  19（46.66）  16 (53.33)  5 (16.67)  5 (16.67)  13.79 (2.18)  13 (43.33)  8 (26.67)  5 (16.67)  1 (3.33)  2(5.71)  1(2.86)  26.65 [21.20, 33.48]  2.03 (0.32)  0.82 (0.06)  11.35 [9.45, 12.95]  1.95 [1.74, 2.41]  0.83 [0.79, 0.92]  18.80 [15.53, 21.03]  1.64 [1.46, 2.09]  0.79 [0.72, 0.86]  291.53 (18.65)  289.50 [276.50, 297.00]  309.00 [282.25, 313.75]  281.30 (17.33)  79.50 [73.25, 83.75]  78.00 [72.50, 86.00]  76.00 [72.25, 80.00]  75.00 [70.50, 78.50]  77.50 [74.00, 81.75]  80.00 [76.00, 83.25]  1.00 [0.30, 5.68]  11.95 [8.91, 16.95]  11.59 (5.06)  14.35 [8.90, 16.50]  10.00 [6.13, 15.78]  0.02 [0.00, 0.05]  0.25 [0.18, 0.35]  0.28 [0.17, 0.38]  0.31 [0.19, 0.37]  0.29 [0.16, 0.41]  0.51 (0.12)  0.19 [0.07, 0.36]  109.00 [103.00, 116.00]  112.83 (24.81)  67.00 [58.50, 76.25]  74.06 (10.30)  3.95 [2.35, 7.18]  17.25 [13.53, 18.28]  17.60 [15.43, 18.28]  16.15 [11.30, 18.02]  15.30 [11.90, 16.80]  0.07 [0.05, 0.19]  0.46 [0.38, 0.48]  0.43 [0.38, 0.47]  0.41 [0.26, 0.46] | 15 (42.86)  20 (57.14)  137.00 [126.00, 143.00]  140（132.25,149.25）  81.26 (5.94)  18（65.71）  12 (34.29)  10 (28.57)  3 (8.57%)  13.94 (2.44)  15 (42.86)  5 (14.29)  8 (22.86)  6 (17.14)  2(6.67)  0(0)  27.30 [21.50, 33.10]  2.19 (0.64)  0.84 (0.10)  12.80 [10.00, 14.80]  2.23 [1.95, 2.57]  0.89 [0.84, 1.01]  17.30 [13.75, 20.20]  1.88 [1.74, 2.11]  0.82 [0.76, 0.89]  295.11 (19.54)  286.00 [278.50, 289.00]  311.00 [290.50, 314.50]  279.54 (22.81)  80.00 [70.00, 85.00]  81.00 [71.00, 87.50]  76.00 [66.00, 81.50]  73.00 [63.50, 77.00]  77.00 [65.50, 81.00]  79.00 [71.50, 83.50]  0.50 [0.30, 1.90]  8.90 [4.45, 13.75]  8.80 (4.55)  9.40 [5.35, 13.35]  7.00 [4.45, 12.70]  0.01 [0.00, 0.05]  0.20 [0.09, 0.33]  0.18 [0.08, 0.27]  0.21 [0.09, 0.31]  0.13 [0.09, 0.29]  0.47 (0.12)  0.15 [0.05, 0.28]  108.00 [91.00, 124.00]  99.40 (32.62)  62.00 [55.50, 68.00]  69.70 (11.84)  2.50 [1.55, 3.85]  16.10 [12.50, 17.95]  15.80 [12.35, 17.65]  13.50 [9.40, 16.60]  12.60 [7.20, 15.35]  0.05 [0.04, 0.09]  0.43 [0.32, 0.47]  0.40 [0.27, 0.46]  0.29 [0.23, 0.42] | 0.744  0.733  0.310  0.304  0.195  0.401  0.455  0.795  >0.999  0.351  0.756  0.112  <0.05  <0.05  0.890  0.196  0.271  0.165  0.065  0.111  0.263  0.117  0.135  0.453  0.353  0.603  0.726  0.859  0.608  0.787  0.285  0.441  0.558  0.300  0.030  0.021  0.014  0.190  0.453  0.132  0.027  0.046  0.008  0.185  0.669  0.622  0.064  0.041  0.118  0.013  0.277  0.104  0.039  0.025  0.027  0.211  0.184  0.048 |
| pSCPPD(T)，mm^-1^ | 0.29 [0.14, 0.36] | 0.37 [0.28, 0.41] | 0.011 |

**Supplementary Table 3****:** The list of λ values considered during cross-validation

|  | Df | %Dev | Lambda |
| --- | --- | --- | --- |
| 1 | 0 | 0.00 | 0.165500 |
| 2 | 1 | 2.76 | 0.137100 |
| 3 | 1 | 4.69 | 0.113600 |
| 4 | 3 | 6.30 | 0.094170 |
| 5 | 5 | 9.03 | 0.078030 |
| 6 | 11 | 13.79 | 0.064660 |
| 7 | 12 | 19.27 | 0.053580 |
| 8 | 12 | 23.71 | 0.044400 |
| 9 | 14 | 27.60 | 0.036790 |
| 10 | 17 | 31.21 | 0.030490 |
| 11 | 21 | 34.62 | 0.025260 |
| 12 | 23 | 38.02 | 0.020930 |
| 13 | 27 | 41.31 | 0.017350 |
| 14 | 31 | 44.49 | 0.014370 |
| 15 | 33 | 47.54 | 0.011910 |
| 16 | 37 | 50.28 | 0.009870 |
| 17 | 39 | 53.16 | 0.008178 |
| 18 | 41 | 55.99 | 0.006777 |
| 19 | 42 | 58.63 | 0.005616 |
| 20 | 43 | 60.87 | 0.004653 |
| 21 | 44 | 63.07 | 0.003856 |
| 22 | 48 | 65.18 | 0.003195 |
| 23 | 49 | 67.24 | 0.002648 |
| 24 | 52 | 69.39 | 0.002194 |
| 25 | 53 | 71.38 | 0.001818 |
| 26 | 53 | 73.26 | 0.001507 |
| 27 | 53 | 75.19 | 0.001248 |
| 28 | 52 | 77.06 | 0.001034 |
| 29 | 55 | 78.94 | 0.000857 |
| 30 | 56 | 80.91 | 0.000710 |
| 31 | 56 | 83.20 | 0.000589 |
| 32 | 55 | 85.56 | 0.000488 |
| 33 | 55 | 87.55 | 0.000404 |
| 34 | 56 | 89.50 | 0.000335 |
| 35 | 56 | 91.25 | 0.000278 |
| 36 | 56 | 92.74 | 0.000230 |
| 37 | 56 | 93.94 | 0.000191 |
| 38 | 56 | 94.98 | 0.000158 |
| 39 | 56 | 95.85 | 0.000131 |
| 40 | 56 | 96.56 | 0.000108 |
| 41 | 56 | 97.14 | 0.000090 |
| 42 | 56 | 97.62 | 0.000074 |
| 43 | 56 | 98.01 | 0.000062 |
| 44 | 56 | 98.34 | 0.000051 |
| 45 | 56 | 98.61 | 0.000042 |
| 46 | 57 | 98.83 | 0.000035 |
| 47 | 57 | 99.02 | 0.000029 |
| 48 | 57 | 99.18 | 0.000024 |
| 49 | 57 | 99.33 | 0.000020 |
| 50 | 57 | 99.42 | 0.000017 |

**Supplementary Table 4:** 7 cases as examples for display in the calculator

|  | Age | Years of education | DBP | SPCA flow rate | pRNFL (I) | Vertical C/D | Prediction results | Actual predictor values |
| --- | --- | --- | --- | --- | --- | --- | --- | --- |
| Sample 1 | 59 | 1 | 90 | 21.9 | 145 | 0.07 | 0.03 | 0 |
| Sample 2 | 59 | 0 | 86 | 18 | 130 | 0.48 | 0.26 | 0 |
| Sample 3 | 71 | 1 | 87 | 11.8 | 151 | 0.38 | 0.11 | 0 |
| Sample 4 | 68 | 0 | 83 | 15.5 | 123 | 0.49 | 0.51 | 1 |
| Sample 5 | 73 | 1 | 66 | 19.9 | 105 | 0.65 | 0.78 | 1 |
| Sample 6 | 78 | 0 | 60 | 25.2 | 98 | 0.66 | 0.98 | 1 |
| Sample 7 | 91 | 0 | 80 | 32.5 | 110 | 0.2 | 0.97 | 1 |
